# A Comparison of Performance of Endotracheal Intubation Using the Levitan FPS Optical Stylet or Lary-Flex Videolaryngoscope in Morbidly Obese Patients

**DOI:** 10.1155/2014/207591

**Published:** 2014-05-20

**Authors:** Tomasz Gaszynski, Monika Pietrzyk, Tomasz Szewczyk, Ewelina Gaszynska

**Affiliations:** ^1^Department of Emergency Medicine and Disaster Medicine, Medical University of Lodz, 90**-**131 Lodz, Poland; ^2^Department of Anesthesiology and Intensive Therapy, Barlicki University Hospital, Medical University of Lodz, Ulice Kopcinskiego 22, 90**-**153 Lodz, Poland; ^3^Department of Gastroenterology, Oncology and General Surgery, Barlicki University Hospital, Medical University of Lodz, 90**-**131 Lodz, Poland; ^4^Department of Hygiene and Health Promotion, Medical University of Lodz, 90**-**131 Lodz, Poland

## Abstract

*Introduction.* The use of videolaryngoscopes is recommended for morbidly obese patients. The aim of the study was to evaluate the Levitan FPS optical stylet (Levitan) vs Lafy-Flex videolaryngoscope (Lary-Flex) in a group of MO patients. *Methods.* Seventy-nine MO (BMI > 40 kg m^−2^) patients scheduled for bariatric surgery were included in the study and randomly allocated to the Levitan FPS or Lary-Flex group. The primary endpoint was time to intubation and evaluation laryngoscopic of glottic view. Anesthesiologists were asked to evaluate the glottic view first under direct laryngoscopy using the videolaryngoscope as a standard laryngoscope (monitor display was excluded from use) and then using devices. The secondary endpoint was the cardiovascular response to intubation and the participant's evaluation of such devices. *Results.* The time to intubation was 8.572.66 sec. versus 5.790.2 sec. for Levitan and Lary-Flex, respectively (*P* < 0.05). In all cases of CL grade >1 under direct laryngoscopy, the study devices improved CL grade to 1. The Levitan FPS produced a greater cardiovascular response than the Lary-Flex videolaryngoscope. *Conclusion.* The Lary-Flex videolaryngoscope and the Levitan FPS optical stylet improve the laryngeal visualization in morbidly obese patients, allowing for fast endotracheal intubation, but Lary-Flex produces less cardiovascular response to intubation attempt.

## 1. Introduction

Obesity is a rising problem amongst population health worldwide. The percentage of obese individuals in the Poland's population is about 12% but in western countries nears 60% [1]. Increased BMI is associated with an increased probability of difficult intubation [2]. This probability is 1.24 times higher with BMI 25–35 kg m^−2^ and 1.42 times higher with BMI ≥ 35 kg m^−2^ when compared to nonobese patients [2]. Other research suggests that the probability of difficult intubation is three times [3] or even six times [4] higher in obese patients compared to nonobese patients.

Laryngoscopy may be difficult in obese patients because of elevated chest diameter giving limited space for the laryngoscope positioning, limited neck mobility, and increased amount of fat tissue in the upper airway, including a larger tongue [5, 6]. Because of these challenges, it is recommended to properly position obese patients for intubation [7].

**Figure 1:**
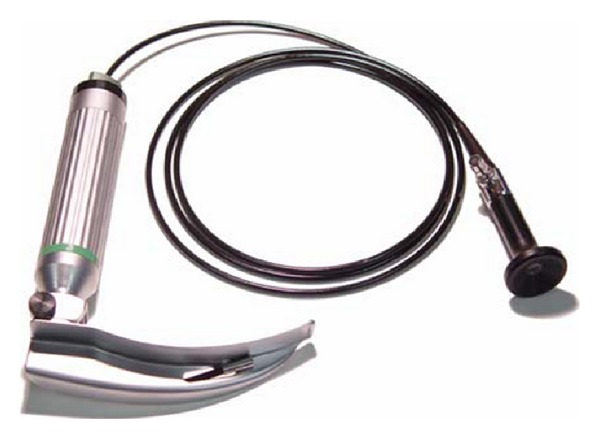
Lary-Flex videolaryngoscope (source: manufacturer marketing materials).

**Figure 2:**
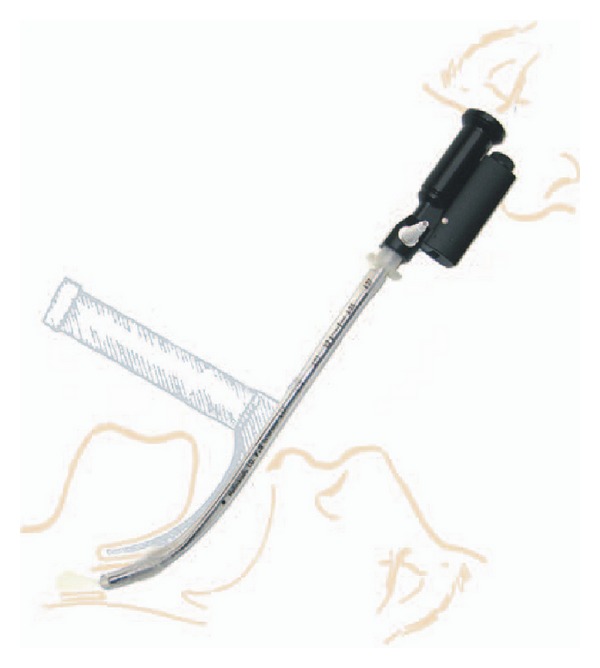
Levitan FPS optical stylet (source: manufacturer marketing materials).

The use of videolaryngoscopes should improve laryngeal view in morbidly obese patients [8, 9]. Although obesity alone is not a risk factor for difficult intubation [10], it is recommended to use videolaryngoscopes as a part of routine practice in anesthesia for morbidly obese patients [11]. The Levitan FPS optical stylet (Clarus Medical, Minneapolis, USA) and Lary-Flex videolaryngoscope (Acutronic, Switzerland) are portable devices for airway management (Figures 1 and 2). A limited number of scientific papers describe clinical experience with these devices in morbidly obese patients. To our knowledge, this is the first prospective randomized study comparing the use of Lary-Flex videolaryngoscope and Levitan FPS in morbidly obese patients.

## 2. Materials and Methods

**Figure 3:**
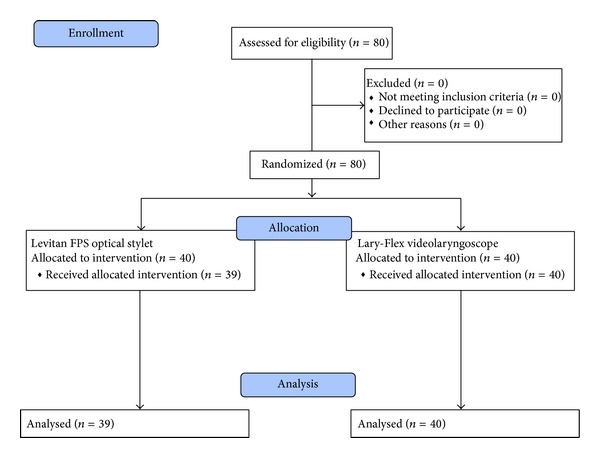
Flow diagram for the study.

The study protocol was approved by the Medical University of Lodz Ethics Committee (Protocol number: RNN/752/10/KB, Chairperson: Professor P. Polakowski, December 14, 2010). Eighty morbidly obese (BMI > 40 kg m^−2^) patients scheduled for bariatric surgery were included into study after receiving written consent. Patients with predicted difficult intubation were excluded from the study: limited mouth opening < 3 cm, Mallampati grade > 3, neck circumflex > 50 cm, and thyromental distance < 6 cm [12]. Patients were randomly allocated to intubate with the Levitan optical stylet or Lary-Flex videolaryngoscope (Figure 3).

For intubation, patients were situated in the head-elevated position [13]. All patients were anesthetized following our institution protocols: induction of anesthesia with propofol 2.0 mg kg^−1^ of corrected body weight; for muscle relaxation rocuronium 0.6 mg kg^−1^ of ideal body weight (IBW); fentanyl 0.05 mg kg^−1^ of IBW. After achieving 100% neuromuscular suppression confirmed by TOF-Watch monitoring, laryngoscopy was performed by various anesthesiologists with ranging degrees of videolaryngoscope experience. All have been working at least few years in the University Hospital Bariatric Centre. Intubation in each patient, allocated to the Levitan group, was facilitated with the use of a laryngoscope and Macintosh-shaped blade. In the Lary-Flex group, intubation was facilitated using videolaryngoscopes as a standard laryngoscope (the monitor display was excluded from use) while also evaluating the glottic view under direct laryngoscopy (C/L 1). Afterwards they were asked to look at the monitor of the videolaryngoscope and facilitate patient intubation (C/L 2). The laryngoscopic conditions were evaluated using the Cormack-Lehane scale. In the Levitan group, anesthesiologists were asked to evaluate the laryngoscopic view using a standard Macintosh blade laryngoscope (C/L 1) and then intubate patient using Levitan FPS optical stylet (C/L 2). Intubation conditions were evaluated using the Krieg scale (Table 1): K1 in the Levitan group using only laryngoscope and in Lary-Flex group using videolaryngoscope as standard laryngoscope and K2 using Levitan FPS and using the whole videolaryngoscope set.

The time from placing the laryngoscope in the hand to insertion of the endotracheal tube was recorded using the same stopwatch in every case and the number of attempts was recorded. Failed intubation was defined as esophageal intubation or intubation attempt taking longer than 30 seconds. Cardiovascular parameters were recorded before intubation (T1) and during intubation (T2) based on the cardiovascular response to intubation. Subsequent to intubation, participants evaluated whether the devices studied aided intubation conditions or not.

## 3. Data Analysis

Our primary endpoint was the time required for successful intubation for each device. The secondary endpoints are evaluation of the efficacy of the study devices in improving glottic visualization and the cardiovascular response to intubations.

Statistical analysis was performed with Statistica 10.0 software (Statsoft, Tulsa, OK, USA). For evaluation of data distribution, the Shapiro-Wilk test was used. The Mann-Whitney *U* test was used for nonpaired categorical and continuous data analysis (time of intubation). For evaluation of numerical scales a Spearman correlation ratio of rang was used. The Chi square test for independent pairs was used with the Yates correction as required (analysis of failed intubation). Post hoc testing was performed with the Fisher LSD test. Kaplan-Meier curves were drawn and a Log-rank test was performed for group comparison. *P* values lower than 0.05 were considered statistically significant.

## 4. Results

**Figure 4:**
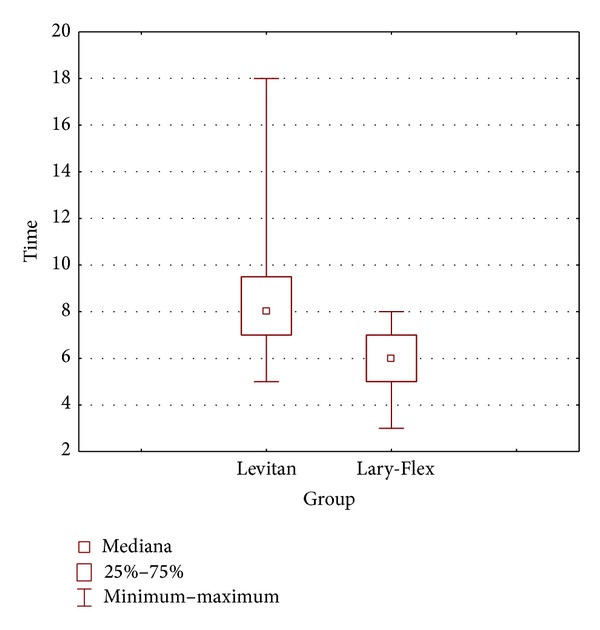
Time for intubation with studied devices (s).

**Figure 5:**
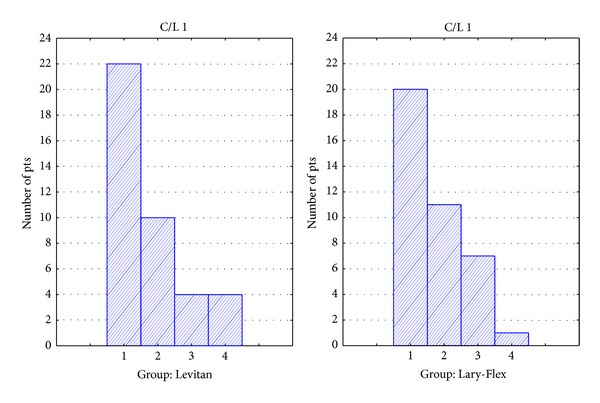
Histogram of evaluation of laryngeal view: Macintosh blade laryngoscope (Levitan group) or Lary-Flex as standard laryngoscope (Lary-Flex group).

**Figure 6:**
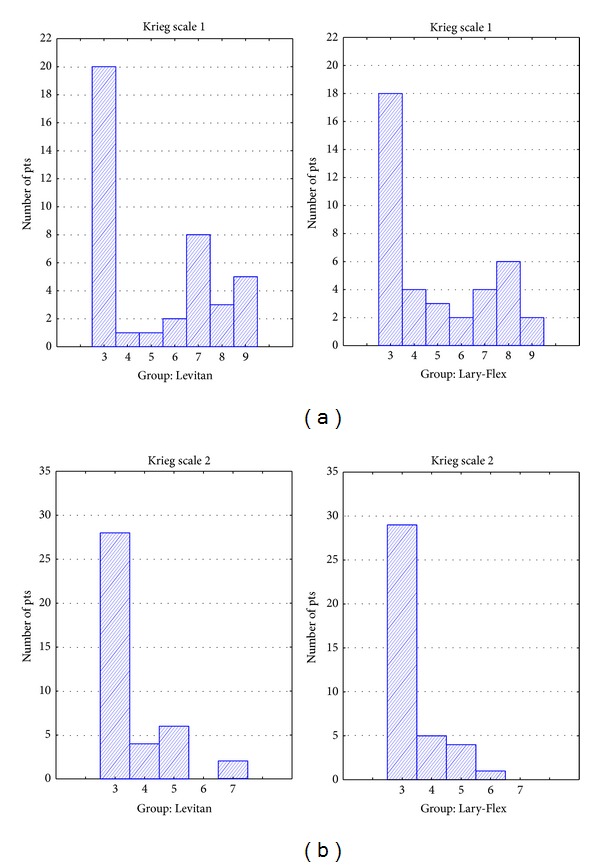
Histograms of evaluation of intubation conditions: Krieg scale 1, conditions using Macintosh blade laryngoscope (Levitan group) or Lary-Flex as standard laryngoscope (Lary-Flex group), Krieg scale 2, conditions using devices.

**Figure 7:**
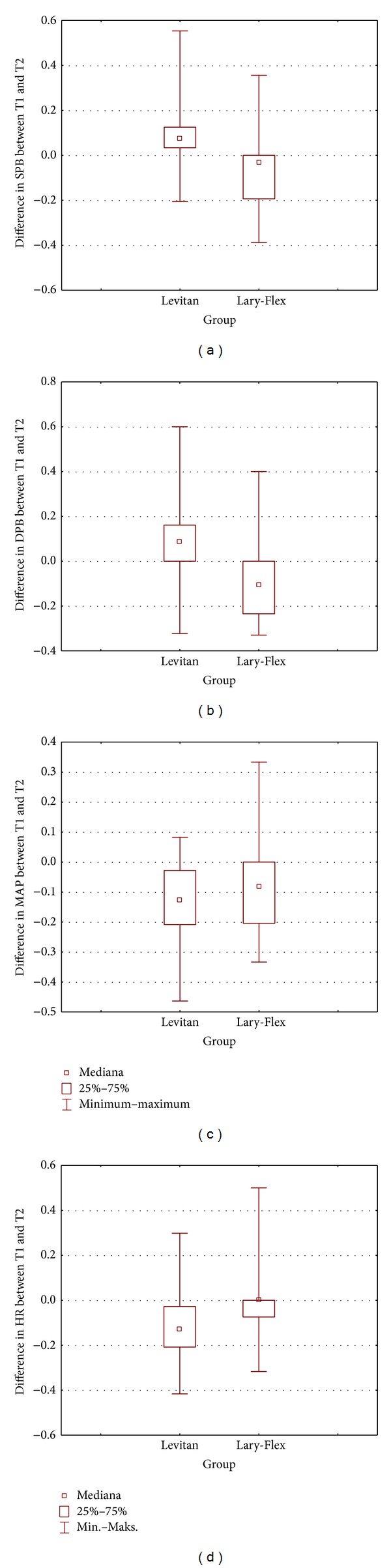
Differences in cardiovascular parameters between time points [%]. T1: preintubation (postinduction), T2: postintubation.

Complete data was collected in 40 patients in the Levitan group and 39 patients in the Lary-Flex group. Demographic data are presented in Table 2. Results of evaluation of preintubation conditions are presented in Table 3. There were no statistical differences in the demographic profiles of groups or in the preintubation tests. For evaluation of intubation conditions, there were no statistically significant differences (Table 4). In the Levitan group, the intubation time was significantly longer but still within acceptable clinical limits—Table 4, Figure 4. Evaluations of the intubation conditions are presented in Figures 5, 6, and 7. In all cases of CL grade > 1 in direct laryngoscopy the study devices improved CL grade to 1. No complications of intubation were observed.

Most of anesthesiologists felt the study devices improved intubation conditions (Table 5). When using the Levitan FPS, the cardiovascular response was significantly larger in comparison to the Lary-Flex videolaryngoscope (Table 6).

## 5. Discussion

There is a wide range of videolaryngoscopes and other airway devices currently available. Videolaryngoscopes can be divided into subgroups: Macintosh-like blades (e.g., C-Mac, McGrath MAC) and modified blades (e.g., McGrath Series 5, Glidescope). The TruView PCD, which we have used, is a laryngoscope with a modified blade. The glottic view is obtained through the optical view tube incorporated into the blade; a video system can be additionally connected. There are also devices with a special channel for the endotracheal tube, for example, AirTraq, Pentax AWS, and King Vision. Nasotracheal AirTraq intubation, which we used, is modified: it has no channel for the tube. A separate group of airway devices are optical stylets. They combine features of fibroscopes and intubation stylets. The intubation tube should be placed over the optical stylet before using. The operator may use them as intubation stylets together with the laryngoscope. When using rigid optical stylets, the operator can look through the ocular (Bonfils, Levitan FPS) like in a fiberscope and observe the entrance to larynx. This allows for location of entrance to larynx in difficult cases and increases the safety of the procedure. The optical stylets are rigid. Only one of the optical stylets, SensaScope, is a rigid optical stylet with a moving tip similar to fiberscopes.

Each device has its advantages and disadvantages. Videolaryngoscopes are advantageous in that they function very similar to that of standard laryngoscopes. As a result, anesthesiologists can quickly master the skill to effectively employ this device. A possible disadvantage to its use is fogging, which may be resolved in a variety of ways. For example, in the case of the C-Mac, applying antifog solution or for AirTraq the device should be turned on 30 seconds prior to use, to warm up the lens. In the case of the TruView PCD, constant oxygen flow to the lens area prevents fogging and removes secretions from the view. Optical stylets have disadvantages similar to fiberscopes: limited view, possible fogging, and further limitation of view by secretions. The advantage to using optical stylets is that they are similar in use to standard intubation introducers; they are easy to use and requiring minimal training.

The general indications for all devices mentioned above are in situations of both predicted and unexpected difficult intubation. Such devices are becoming more frequently employed in cases of standard patients because they provide optimal prevention of possible intubation injuries in comparison to standard laryngoscopes. In the case of predicted intubation difficulties (like in the case of morbidly obese patients) the fiberscope is preferred; however, the modern airway devices can be a good alternative. For the anesthesiologists who are using the new devices during every day practice they create new opportunities to manage difficult patients in an effective way, including morbidly obese patients [1].

The primary goal was to improve the time required for intubation. As evidenced with use of the Lary-Flex videolaryngoscope, intubation times were slightly improved. Though intubation conditions were comparable, the cardiovascular response to intubation attempts was smaller while employing the Lary-Flex videolaryngoscope. However, the majority of participants stated that both devices seemed to improve intubation conditions.

The use of videolaryngoscopes in morbidly obese patients not only improves glottic view but also makes intubation efforts easier and less traumatic [14, 15]. Therefore, although obesity is not associated with a higher probability of difficult intubation, endotracheal intubation in morbidly obese patients success requires skill and usually more strength. In our study we confirmed previous reports results that standard Mallampati evaluation cannot predict an increased Cormack-Lehane score in morbidly obese patients [16], and in this group of patients it is justified to use videolaryngoscopes as a standard practice. The Lary-Flex videolaryngoscope and Levitan FPS optical stylet proved to be very good, effective, and easy to use even for anesthesiologists with limited experience using videolaryngoscopes. There is no other study evaluating the Lary-Flex or Levitan FPS in morbidly obese patients. In the study describing the use of V-Mac videolaryngoscope (previous version of C-Mac) in morbidly obese patients, Maassen et al. demonstrate similar results to ours: intubation time of 17 sec. but more intubation efforts (average 1.4) [17]. We evaluated the C-MAC videolaryngoscope (Storz, Germany) in morbidly obese patients and we found that it improved the laryngeal view [9]. In this study and the presented study, all cases of the intubation were successful within the recommended time for intubation attempts, which is especially important in morbidly obese patients, in whom desaturation is faster than that in nonobese patients [18]. Maassen et al. compared three videolaryngoscopes: Storz V-MAC, Glidescope Ranger, and McGrath in MO patients [17]. They concluded that the Storz V-MAC was better than the other devices evaluated for intubation of MO patients. Dhonneur et al. proved that the use of the X-Lite Videolaryngoscope improved intubation conditions in MO [15]. Ndoko et al. evaluated the AirTraq in MO and demonstrated similar results to those concluded within our study; AirTraq is a useful device in this group of patients [19]. We evaluated the AirTraq in morbidly obese patients and found that it improves intubation conditions in such patients [20]. Dhonneur et al. also validate similar results [15, 21]. Andersen et al. compared the Glidescope videolaryngoscope and the standard laryngoscope [22]. Slightly longer intubation times were attained; however, significantly better intubation difficulty scale scores were also achieved with use of the Glidescope. The use of a videolaryngoscope is justified in patients in whom the probability of difficult intubation is increased because of coexisting diabetes mellitus, a common comorbidity in morbidly obese patients [23]. Videolaryngoscopes may be used instead of fibroscopes for awake intubation [24, 25].

The cardiovascular response to videolaryngoscopy is smaller when compared to the use of standard Macintosh laryngoscopes in morbidly obese patients [17]. The cardiovascular response while using the Levitan FPS was not previously evaluated in such patient population. As noted in other studies, Levitan FPS should provoke less of a cardiovascular response comparing to standard laryngoscopy [26]. Some authors compared fiberoptic intubation with the use of Bonfils optical stylets. They concluded that both devices require a similar time for successful orotracheal intubation and cause a similar magnitude of hemodynamic response [27]. As evidenced in our study, the response to intubation using the Levitan FPS device was greater than that of the Lary-Flex videolaryngoscope. This may be justified as the Macintosh laryngoscope was employed while using the Levitan FPS device.

The cardiovascular response to the videolaryngoscopy may be similar to insertion of supraglottic devices in morbidly obese patients [28]. By reducing the stress response, videolaryngoscopes may prove advantageous to the standard laryngoscope in obese patients. Although transitory hypertension and tachycardia are probably of little clinical consequence in healthy individuals, they may be a matter of concern in patients with known, or at risk of, cardiovascular disease such as obese patients [28]. Smaller release of stress hormones may influence the postoperative outcome [28]. The response from the cardiovascular system is smaller for videolaryngoscopes than that with standard laryngoscopy [29]. Although fiberoptic intubation in morbidly obese is still recommended, there is no evidence that this technique is superior to videolaryngoscopy [30]. On the contrary, some studies demonstrate that videolaryngoscopy is good alternative to fiberoptic intubation in morbidly obese patients [30].

Both the Lary-Flex videolaryngoscope and the Levitan FPS optical stylet proved to be very effective for intubation of morbidly obese patients. Therefore, we suggest that videolaryngoscopes and optical stylets should be recommended for routine practice in anesthesia for morbidly obese patients.

## 6. Conclusion

Both devices, the Lary-Flex videolaryngoscope and the Levitan FPS optical stylet, improve the laryngeal view in morbidly obese patients and allowed for efficient endotracheal intubation. However, the Lary-Flex videolaryngoscope produced less cardiovascular response to endotracheal intubation attempt.

## Figures and Tables

**Table 1:** Krieg scale.

Score	1	2	3	4

Laryngoscopy	Easy	Good	Difficult	Impossible

Vocal cords	Open	Move	Closing	Closed

Coughing reflex	Absent	Diaphragm move	Weak	Strong

Evaluation of intubation conditions: 3-4: ideal; 5-6: good; 8–10: poor; 10–12: difficult.

**Table 2:** Demographic data of studied groups.

Group	Parameter	*N*	Mean	95% CI		Median	Minimum	Maximum	Q1	Q3	SD	SE	P group Levitan versus Lary-Flex
Levitan	Age (yrs)	40	40.10	36.57	43.63	39.00	19.00	60.00	32.50	48.50	11.05	1.75	0.307822
	Weight (kg)	40	126.03	119.43	132.62	123.00	100.00	200.00	109.50	139.00	20.63	3.26	0.968712
	Height (cm)	40	168.03	165.21	170.84	165.50	154.00	190.00	163.00	172.00	8.80	1.39	0.606690
	BMI (kg/m^2^)	40	44.58	42.73	46.42	44.14	35.15	57.75	40.28	49.06	5.77	0.91	0.821564

Lary-Flex	Age (yrs)	39	38.05	34.35	41.75	35.00	20.00	64.00	30.00	47.00	11.42	1.83	
	Weight (kg)	39	124.90	119.78	130.02	125.00	95.00	160.00	115.00	132.00	15.79	2.53	
	Height (cm)	39	167.31	164.47	170.15	165.00	152.00	190.00	162.00	170.00	8.76	1.40	
	BMI (kg/m^2^)	39	44.99	43.28	46.70	43.82	38.75	60.97	40.77	49.08	5.28	0.85	

95% CI: confidence interval; Q1: quartile 1; Q3: quartile 3; SD: standard deviation; SE: standard error.

**Table 3:** Evaluation of preintubation conditions data of studied groups.

Group	Parameter	*N*	Mean	95% CI		Median	Minimum	Maximum	Q1	Q3	SD	SE	*P* value Levitan versus Lary-Flex
Levitan	Mallampati scale	40	1.28	1.11	1.44	1.00	1.00	3.00	1.00	1.50	0.51	0.08	0.960896
	Thyromental distance	40	6.78	6.57	7.00	6.70	6.0	8.10	6.25	7.30	0.67	0.11	0.705785

Lary-Flex	Mallampati scale	39	1.28	1.12	1.45	1.00	1.00	3.00	1.00	2.00	0.51	0.08	
	Thyromental distance	39	6.84	6.64	7.04	6.90	6.0	8.00	6.30	7.30	0.61	0.10	

95% CI: confidence interval; Q1: quartile 1; Q3: quartile 3; SD: standard deviation; SE: standard error.

**Table 4:** Evaluation of intubation conditions data of studied groups.

Group	Parameter	*N*	Mean	95% CI		Median	Minimum	Maximum	Q1	Q3	SD	SE	*P* value Levitan versus Lary-Flex
Levitan	Intubation time	40	8.57	7.72	9.43	8.00	5.00	18.00	7.00	9.50	2.66	0.42	*0.000000 *
	Number of attempts	40	1.08	0.99	1.16	1.00	1.00	2.00	1.00	1.00	0.27	0.04	0.705785
	C/L 1	40	1.75	1.43	2.07	1.00	1.00	4.00	1.00	2.00	1.01	0.16	0.898564
	C/L 2	40	1.00			1.00	1.00	1.00	1.00	1.00	0.00	0.00	1.000000
	K1	40	5.15	4.39	5.91	3.50	3.00	9.00	3.00	7.00	2.38	0.38	0.848359
	K2	40	3.60	3.25	3.95	3.00	3.00	7.00	3.00	4.00	1.08	0.17	0.651942

Lary-Flex	Intubation time	39	5.79	5.40	6.19	6.00	3.00	8.00	5.00	7.00	1.22	0.20	
	Number of attempts	39	1.03	0.97	1.08	1.00	1.00	2.00	1.00	1.00	0.16	0.03	
	C/L I	39	1.72	1.44	2.00	1.00	1.00	4.00	1.00	2.00	0.86	0.14	
	C/L II	39	1.00	—	—	1.00	1.00	1.00	1.00	1.00	0.00	0.00	
	K1	39	4.90	4.19	5.60	4.00	3.00	9.00	3.00	7.00	2.17	0.35	
	K2	39	3.41	3.16	3.66	3.00	3.00	6.00	3.00	4.00	0.79	0.13	

95% CI: confidence interval; Q1: quartile 1; Q3: quartile 3; SD: standard deviation; SE: standard error; C/L 1: evaluation of laryngoscopic view in Cormack-Lehane scale at beginning of intubation: for Levitan group using only laryngoscope, for Lary-Flex group using videolaryngoscope as standard laryngoscope; C/L 2: evaluation of laryngoscopic view using Levitan FPS or looping on monitor of Lary-Flex videolaryngoscope. Intubation conditions were evaluated using Krieg scale K1 in Levitan group using only laryngoscope, in Lary-Flex group using videolaryngoscope as standard laryngoscope and K2 using Levitan FPS and using whole videolaryngoscope set.

**Table 5:** Evaluation of participants opinion of potential improvement of intubation conditions.

Group	Improvement of intubation conditions	
	No	Yes
Levitan	45.00%	55.00%
Lary-Flex	48.72%	51.28%

**Table 6:** Cardiovascular response to intubation attempts.

Group	Parameter		*N*	Mean	95% CI		Median	Minimum	Maximum	Q1	Q3	SD	SE	*P* value Levitan versus Lary-Flex
Levitan	SPB	T1	40	119.03	114.14	123.91	119.00	94.00	143.00	105.50	130.50	15.29	2.42	0.197238
		T2	40	129.07	124.23	133.92	126.00	108.00	160.00	114.00	143.00	15.15	2.40	*0.000000 *
	DBP	T1	40	74.25	70.81	77.69	72.50	50.00	94.00	66.50	80.50	10.76	1.70	*0.007321 *
		T2	40	81.03	78.07	83.98	80.00	63.00	96.00	74.00	90.00	9.24	1.46	*0.000000 *
	MAP	T1	40	102.88	97.86	107.89	101.00	71.00	132.00	94.00	110.00	15.69	2.48	***0.000004***
		T2	40	88.50	85.05	91.95	88.00	66.00	110.00	81.00	97.00	10.78	1.70	***0.000000***
	HR	T1	40	88.53	83.42	93.63	85.00	54.00	122.00	77.50	96.00	15.95	2.52	*0.000000 *
		T2	40	75.88	71.89	79.86	77.50	54.00	105.00	65.00	85.00	12.46	1.97	*0.000029 *

Lary-Flex	SBP	T1	39	113.97	106.40	121.55	119.00	73.00	165.00	94.00	130.00	23.38	3.74	
		T2	39	102.51	97.31	107.71	101.00	71.00	143.00	94.00	118.00	16.04	2.57	
	DBP	T1	39	66.05	60.48	71.62	61.00	40.00	98.00	52.00	83.00	17.19	2.75	
		T2	39	58.69	54.68	62.70	58.00	42.00	98.00	50.00	64.00	12.37	1.98	
	MAP	T1	39	81.18	75.51	86.85	78.00	54.00	112.00	66.00	98.00	17.48	2.80	
		T2	39	73.13	68.83	77.42	73.00	52.00	112.00	64.00	78.00	13.25	2.12	
	HR	T1	39	64.41	61.17	67.65	66.00	48.00	82.00	53.00	72.00	9.98	1.60	
		T2	39	63.92	61.26	66.58	66.00	51.00	77.00	55.00	70.00	8.20	1.31	

T1: preintubation (postinduction); T2: postintubation.
